# Sprouty 1 is associated with stemness and cancer progression in glioblastoma

**DOI:** 10.1016/j.ibneur.2022.07.003

**Published:** 2022-07-21

**Authors:** Seo-Young Park, Hang Yeon Jeong, Don Carlo Batara, Suk Jun Lee, Jeong-Yong Cho, Sung-Hak Kim

**Affiliations:** a Animal Molecular Biochemistry Laboratory, Department of Animal Science, College of Agriculture and Life Sciences, Chonnam National University, Gwangju, the Republic of Korea; b Research Group of Aging Metabolism, Korea Food Research Institute, Wanju, the Republic of Korea; c Department of Biomedical Laboratory Science, College of Health & Medical Sciences, Cheongju University, Chungbuk, the Republic of Korea; d Department of Food Science and Technology, Chonnam National University, Gwangju, the Republic of Korea

**Keywords:** SPRY1, Glioblastoma multiforme, Glioma stem cell, GBM

## Abstract

Glioblastoma multiforme (GBM) is the most severe type of human brain tumor, with a poor prognosis and a low survival rate. GBM is composed of a variety of cell types, including glioma stem-like cells (GSCs), which attribute to its therapeutic resistance (Boyd et al., 2020). Sprouty1 (SPRY1) was first identified as a receptor tyrosine kinases (RTK) signaling mediator in a mammalian cell (Christofori, 2003), however, its role in GBM is unknown. Therefore, the goal of this study was to investigate the role of SPRY1 in the stemness and aggressiveness of GSCs. The mRNA expression levels of *SPRY1* were confirmed using quantitative reverse transcription PCR (RT-qPCR) in normal human astrocytes (NHA), glioma cells, and glioma stem cells. SPRY1 expression was inhibited in glioma stem cells using small interference RNA (siRNAs) to examine its role in cell proliferation and tumorsphere formation. Bioinformatics analyses were also employed to investigate the association of *SPRY1* expression with patient survival, tumor grade, and subtypes publicly available datasets. We demonstrated that SPRY1 is highly expressed in glioma stem cells than in NHA, glioma cells, and differentiated glioma stem cells. siRNA-mediated downregulation of *SPRY1* expression decreased the stemness and self-renewal ability in GSC11. Bioinformatics results showed that high SPRY1 expression correlates with poor overall survival in glioma patients. Our findings suggest that SPRY1 contributes to the stemness and aggressiveness of GBM.

## Highlights

• Glioblastoma (GBM) is the most aggressive cancer type in human brain.
• Glioma stem cell (GSC) is a small population in GBM, associated therapy resistance and recurrence.
• *Sprouty1* expression associated with GBM grade and patient survival.
• *Sprouty1* highly expressed in GBM cell lines and GSC.
• Suppression of SPRY1 expression downregulated GSC proliferation and tumorsphere formation.

## 1 Introduction

Glioblastoma (GBM) is the most aggressive and common primary brain tumor and is designated Grade IV by the World Health Organization (WHO) (Boyd et al., 2020). Despite advancements in surgical resection, radiation, and concurrent chemotherapy, there is no significant improvement in GBM patients' survival. Epidemiologic data shows that GBM patients have less than a 5 % survival rate within 5 years and 14.6 months overall median survival among adults (Jeong et al., 2020). GBM is composed of a population of glioma stem cells (GSCs), which are capable of self-renewal and differentiation, implying a role in tumor recurrence and therapeutic resistance (Jeong et al., 2020). Hence, therapeutic targets in GSCs are a focus of interest to improve the GBM outcome.

Sprouty, a family member of Sprotuy1 (SRPY1), is a modulator of Receptor Tyrosine Kinase (RTK) signaling in normal development and disease (Masoumi-Moghaddam et al., 2015). RTK signaling provides growth signals to cells through phosphorylating substrates by cascade interaction specific ligands such as basic fibroblast growth factor (bFGF) and epidermal growth factor (EGF). These ligands have been used to maintain self-renewal activities in stem cells during cell culture. ﻿﻿Several studies indicate the role of Spry1 in carcinogenesis, both as a tumor suppressor and an oncogene depending on the cellular context (Christofori, 2003). SPRY1 and SPRY2 were shown to be downregulated in a variety of cancers, including liver, prostate, and lung tumors, implying a unique tumor-suppressive function (Montico et al., 2020).﻿ Conversely, SPRY1′s high expression in tumors harboring Ras/Raf mutations to tumor progression (He et al., 2016) suggests its role in cancer malignancy. For example, SPRY1 expression, which is elevated by oncogenic RAS signaling in rhabdomyosarcoma tumors, is important for cell proliferation and survival *in vitro* and *in vivo* (Schaaf et al., 2010)﻿. SPRY1 may also play a pathological role in GBM malignancy and progression; however, this is yet to be confirmed.

Here, we found that SPRY1 is highly expressed in glioma stem cells, compared to glioma cell lines and non-tumor cells. We also discovered that SPRY1 is co-expressed with stem cell markers and is more elevated in GSCs than in differentiated cells which correlates with their ability to develop as tumorspheres. Further, we showed that knockdown of SPRY1 decreased GSC proliferation and sphere-forming ability. Finally, we confirmed that the expression of SPRY1 is highly enriched in the classical and mesenchymal subtypes, and its overexpression is associated with poor patient survival in the public datasets.

## 2 Materials and methods

### 2.1 Cell culture

Normal human astrocytes (NHA) were cultured in astrocyte media (Welgene, South Korea) supplemented with10 % fetal bovine serum (FBS; HyClone, Thermo Fisher Scientific, USA), Astrocyte Growth Supplement (AGS; Welgene, South Korea), and 1 % penicillin-streptomycin (P/S; Welgene, South Korea). Glioma cells (U87MG, A172, and A1207) were maintained in Dulbecco's modified Eagle's medium (DMEM/F-12; Welgene, South Korea) supplemented with 10 % FBS and 1 % P/S. The patient-derived glioma stem cells (GSC267, GSC11, GSC23, and GSC20) were sourced from The University of Texas MD Anderson Cancer Center. GSCs were cultured in serum-free neurobasal media (NBE) composed of DMEM/F-12, 2 % B-27 (Glico, Thermo-Fisher, USA), 1 % P/S, 20 ng/ul basic fibroblast growth factor (bFGF; R&D Systems), and 20 ng/ul basic epidermal growth factor (EGF; R&D Systems). For GSC differentiation, GSC11 and GSC23 cells were cultured for 10 days in DMEM/F-12 supplemented with 10 % FBS.

### 2.2 RT-qPCR

Following the manufacturer's manual, total RNA was obtained using RiboEx reagent (GeneAll, South Korea) and purified using a Hybrid-R kit (GeneAll, South Korea). The ReverAid First-Strand cDNA Synthesis Kit (Thermo Fisher Scientific, USA) was used to generate complementary DNA (cDNA) from 1 μg of total RNA. Real-time PCR was performed using TB Green Premix Ex Taq (Takara Bio, Japan) and CFX96 real-time PCR (Bio-Rad Laboratories, USA). The cycle threshold (Ct) values were performed to analyze the RT-qPCR results, which were then quantified using the 2^-∆∆ct^ method. Primer sequences used for RT-qPCR amplification (5′ to 3′): SPRY1 Forward(F): GAAAGAGGACCTGACACAGCAC and Reverse (R): CTCTCAGCAGAGCAAAGGCACT; CD133: F: CAGGTAAGAACCCCGATCAA and R: TCAGATCTGTGAACGCCTTG; CD15: F: TTGGGACCTCCTAGTTCCAC and R: TGTAAGGAAGCCACATTGGA; GFAP: F, GGAACATCGTGGTGAAGACC and R: AGAGGCGGAGCAACTATCCT; S100B: F, TCAAAGAGCAGGAGGTTGTG and R: TCGTGGCAGGCAGTAGTAAC; TUBB3: F, AGTGTGAAAACTGCGACTGC and R: ACGACGCTGAAGGTGTTACT; 18 S (control): F: CAGCCACCCGAGATTGAGCA and R: TAGTAGCGACGGGCGGTGTG.

### 2.3 Data set for SPRY1 expression and survival analysis

Repository for Molecular Brain Neoplasia Data (REMBRANDT), and Ivy Glioblastoma Atlas Project datasets were used for the analysis of SPRY1 expression. REMBRANDT dataset was obtained from GlioVis (https://gliovis.bioinfo.cnio.es/).

### 2.4 Small interfering RNA (siRNA) transfection

Small interfering RNAs (siRNA) for inhibiting the SPRY1 transcript, were purchased from Bioneer (Daejeon, South Korea). The siRNA sequence (5′ to 3′) was: siSPRY1: F – GAGAGAGAUUCAGCCUACU; and R - AGUAGGCUGAAUCUCUCUC. A scrambled-siRNA (silencer negative control, SN-1002; Bioneer, Daejeon, South Korea), was used to determine the effects of siRNA delivery and has no sequence similarity, and does not target human gene sequences. To knockdown SPRY1, GSC11 were seeded in a 6-well plate at a density of 5 × 10^5^ cells/well, and after 1 day, it was transfected with 50nmol/L of SPRY1 siRNA or scrambled-siRNA with Lipofectamine® RNAiMAX Reagent (Invitrogen, USA) and incubated for 2 days.

### 2.5 Tumorsphere assay

siRNA transfected GSCs (5 ×10^5^ cells/well) were incubated for 5 days at 5 % CO_2_ and a temperature of 37 ℃ without disturbing. After 5 days of incubation, spheres were counted 3 times using a digital microscope (Logos Biosystems, Anyang-si, South Korea). The counted spheres were based on more than 100 µm in diameter.

### 2.6 Cell viability

To test the siRNA transfection effect on cell viability, GSCs were seeded in 12-well plates of coated laminin with a density of 1 × 10^5^ cells/well, and incubated at 5 % CO^2^ and temperature of 37 ℃. After siRNA transfection, cell number was counted using the ADAM-MC cell counter (NanoEntek, South Korea).

### 2.7 Statistical analysis

Statistical analyses were performed on Microsoft Excel 2016 (Microsoft Corporation, USA), and GraphPad Prism 5 software (GraphPad Software Inc. USA). The *p-value* < .05 indicated statistical significance. A two-tailed *t-test* was used to compare significant quantitative differences among groups. The survival distribution was calculated according to the Kaplan-Meier survival analysis which was compared with stratified groups by the median value as the cutoff.

## 3 Results

### 3.1 SPRY1 mRNA expression is correlated with patients' poor survival

**Fig. 1:**
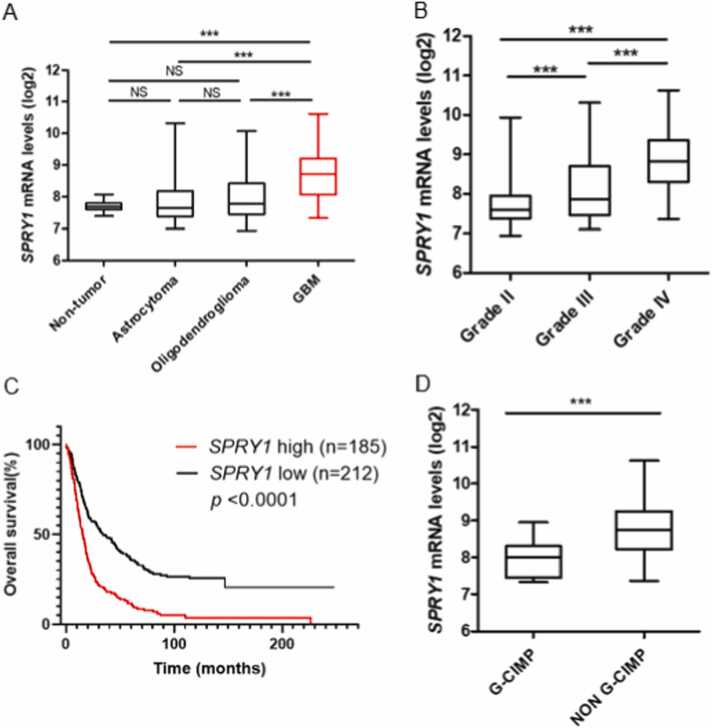
SPRY1 is highly expressed in GBM, A. SPRY1 expression in GBM was compared using RNA-seq of REMBRANDT dataset. Data are mean ± SEM (nontumor, n = 28; astrocytoma, n = 147; oligodendroglioma, n = 67; GBM, n = 219). * **P < .001. B. SPRY1 expression compared GBM grades (Grade I and II, n = 100; Grade III, n = 85; Grade IV, n = 130). * **P < .001. C. Using microarray of REMBRANDT dataset, Kaplan-Meier survival curves of 397 GBM patients (SPRY1 high n = 185, SPRY1 low n = 212, P < .0001). D. SPRY1 expression compared IDH-mutant GBM.

We correlated the clinical relevance of SPRY1 mRNA levels in various GBM histology and grades using a REMBRANDT dataset. SPRY1 was more expressed in GBM subtypes compared with those in oligodendroglioma and astrocytoma (Fig. 1A). Interestingly, SPRY1 mRNA expression was correlated with glioma grades (Fig. 1B), and higher levels of SPRY1 patients showed a shorter survival rate than those with lower levels of SPRY1 (p. value <.0001) (Fig. 1C). G-CIMP is a new classification that approves the molecular classification of isocitrate dehydrogenase (IDH) mutational status in glioma. IDH-mutant glioma means cytosine-phosphate-guanine (CpG) island methylator phenotype (G-CIMP). Non-G-CIMP patients have a loss of DNA methylation, which enhances cancer proliferation, than G-CIMP-high patients (Verhaak et al., 2010). Hence, non-G-CIMP patients have shorter overall survival. SPRY1 mRNA expression level is high in Non-G-CIMP patients (Fig. 1D). These findings indicate that SPRY1 expression is related to poor survival in glioma patients.

### 3.2 Expression levels of SPRY1 are highly expressed in glioma stem cells

**Fig. 2:**
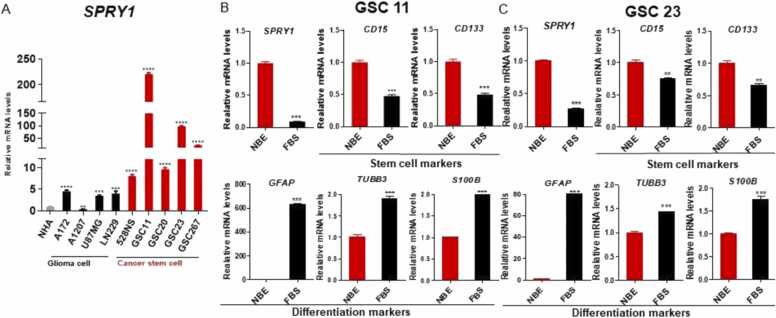
SPRY1 is highly expressed in glioma cells and glioma stem cells than in normal astrocytes. A. Real-time qPCR analysis of *SPRY1* mRNA expression in normal human astrocyte (NHA), glioma cells, and glioma stem cells. NHA *vs.* glioma cells or glioma stem cells. B and C. mRNA expression of *SPRY1*, stem cell markers, and differentiation markers in NBE cultured and FBS cultured GSC11 (B) and GSC23(C). NBE *vs.* FBS in GSC11 and GSC23. Data are means ± SEM (n = 3). Data are means ± SEM (n = 3). *P < .05, * **P < .001, * ** *P < .0001.

Previously, it was reported that undifferentiated GSCs culture in NBE media indicates more heterogeneous morphology, strong tumorigenic potential, and heterogeneous and indefinite self-renewal ability compared to the serum cultured differentiated GSCs (Lee et al., 2006). We compared the SPRY1 mRNA expression between GBM cell lines and GSCs in vitro. Surprisingly, SPRY1 mRNA expression is highly upregulated in GSCs (GSC11, 20, 23, and 267) than in other glioma cells (A172, A1207, LN229, and U87MG) and normal astrocytes, NHA (Fig. 2A). We confirmed a strong downregulation of SPRY1 mRNA expression in GSCs cultured under the FBS condition (Fig. 2B). FBS condition-cultured GSCs, showed strong upregulation of differentiation markers (GFAP, and TUBB3), and downregulation of stemness markers (CD133, and CD15) (Fig. 2B). These data indicate that SPRY1 expression in GSC is associated with GSC stemness.

### 3.3 Silencing of SPRY1 inhibits GSC tumorsphere formation

**Fig. 3:**
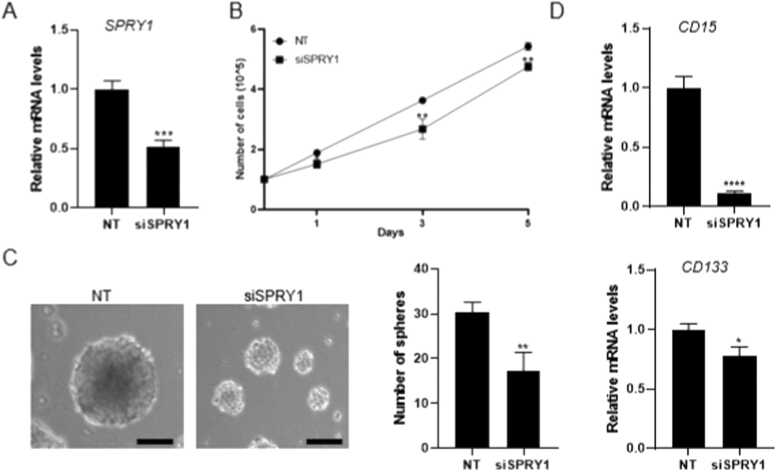
Knockdown of SPRY1 inhibits the stemness of GSCs, A**.** Realtime-qPCR indicated SPRY1 knockdown efficiency using siRNA transfection in GSCs. B. Measurement of cell proliferation rate in NT (Non-targeting siRNA) and siSPRY1. *P < .05, * *P < .01. C. Effect of SPRY1 knockdown on neurosphere formation in GSC11. Data are means ± SEM (n = 3). * *P < .01. Scale bars represent 100 µm. D. Effect of SPRY1 knockdown on stem cell markers, *CD15* and *CD133* mRNA expression in GSC11. Data are means ± SEM (n = 3). *P < .05, * ** *P < .0001.

We hypothesized that SPRY1 function in GSC may contribute to GSC stemness. Therefore, we designed small interfering RNAs (siRNA) to silence the SPRY1. RT-qPCR experiment indicated that siSPRY1 inhibits SPRY1 mRNA levels in GSC11 by 50 % (Fig. 3A). To test the SPRY1 knockdown effect on GSC11 proliferation, cells were counted on days 1, 3, and 5 after siRNA transfection. Data showed that siSPRY1 significantly decreased GSC11 proliferation compared to the control (Fig. 3B). Similarly, knockdown of SPRY1 in GSC11 downregulated tumorsphere formation compared to the control group, indicating that SPRY1 is required for the self-renewal ability of GSC11 (Fig. 3C). Also, the knockdown of SPRY1 downregulated the expression levels of GSC stemness markers (CD133 and CD15) (Fig. 3D). As such, these results prove that SPRY1 expression is important in the self-renewal and proliferation of GSCs.

### 3.4 SPRY1 positive correlation genes were associated with the stemness pathway

**Fig. 4:**
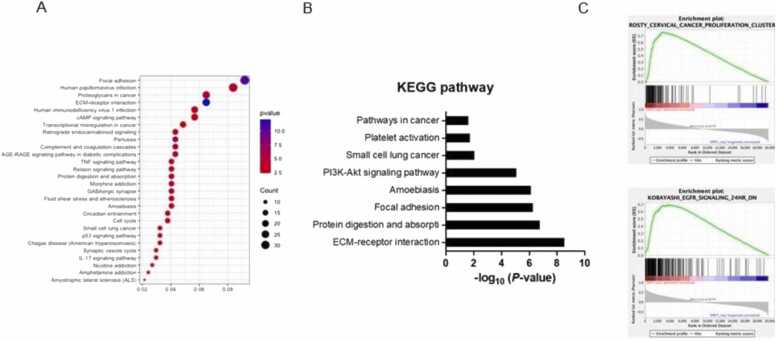
SPRY1 positively correlates with genes associated with cancer stem cell stemness pathway. A. Upregulated genes correlated with high expression of SPRY1 analysis to Gene Ontology analysis (GO analysis) in REMBRANDT dataset. B. Kyoto Encyclopedia of Genes and Genomes (KEGG) pathway analysis in REMBRANDT microarray dataset. C. Gene Set Enrichment Analysis (GSEA) in REMBRANDT; Representative enriched pathways in SPRY1 positive correlated samples.

In the REMBRANDT dataset, we sorted 60 genes whose expression patterns correlated with *SPRY1* expression. Sorted genes were analyzed on the website (http://david.ncifecrf.gov/). GO analysis results indicated that up-regulated DEGs interestingly abounded in focal adhesion, ECM-receptor interaction, and cAMP signaling pathway (Fig. 4A). KEGG pathway analysis showed SPRY1-related genes were involved in ECM-receptor interaction, focal adhesion, and PI3K-Akt signaling pathway (Fig. 4B). GSEA analysis and SPRY1 showed a positive correlation to genes associated with EGFR signaling, and cancer proliferation (Fig. 4C). These findings show that SPRY1 expression is correlated with genes responsible for GBM progression.

### 3.5 Classical and mesenchymal subtypes and GBM vascular region were associated with SPRY1 expression

**Fig. 5:**
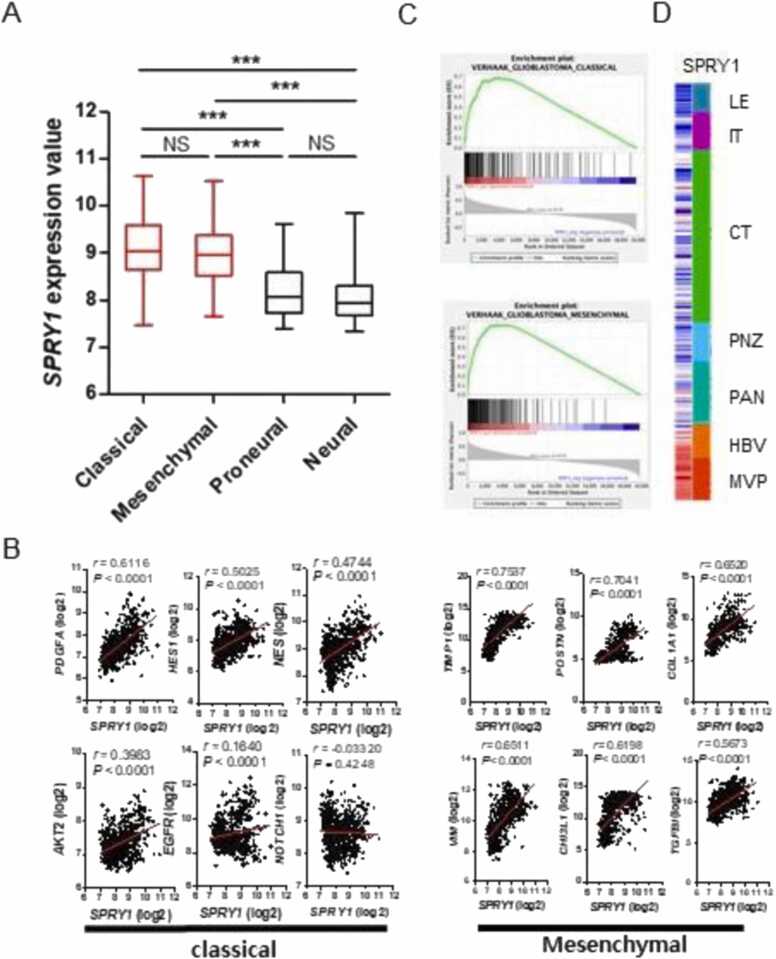
SPRY1 correlated GBM subtype and region. A. Comparison of SPRY1 mRNA levels among the groups with GBM subtypes (Classical n = 99, Neural n = 39, Mesenchymal n = 37, Proneural n = 44) * ** P < .001. B. The graph of SPRY1 between GBM subtypes markers correlation in REMRANDT dataset. C. GSEA analysis of REMBRANDT dataset, SPRY1 positive samples correlated with GBM subtypes. D. Heatmap of SPRY1 expression signature correlated with GBM region in the Ivy GAP RNAseq dataset. CT, cellular tumor; IT, infiltration tumor; LE, leading edge; MVP, microvascular proliferation; HBV, hyperplastic blood vessels; PAN, pseudopalisading cells around necrosis; PNZ, perinecrotic zone.

Neural, proneural, classical, and mesenchymal have been identified in GBM molecular classification based on RNA expression analysis of 300 or more GBM patient tissues. The subtype correlated with tumor histological features, molecular alterations, and distinct clinical outcomes (Verhaak et al., 2010). To examine SPRY1 and GBM subtype correlation, we sorted SPRY1 enriched patient cohorts in the REMBRANDT dataset. SPRY1 mRNA levels were enhanced in classical and mesenchymal subtypes (Fig. 5A). Furthermore, gene expressions of markers in classical (PDGFA, HES1, NES, AKT2, EGFR, and NOTCH1) and mesenchymal (TIMP1, POSTN, COL1A1, VIM, CHI3L1, and TGFBI) were significantly correlated with SPRY1 mRNA levels (Fig. 5B). Furthermore, in Gene Set Enrichment Analysis (GSEA), SPRY1 was positively correlated to genes that are associated with classical and mesenchymal subtypes (Fig. 5C). The Ivy Glioblastoma Atlas Project dataset contains mRNA expression profiles of different tumor areas: cellular tumor (CT), infiltrating tumor (IT), leading-edge (LE), microvascular proliferation (MVP), hyperplastic blood vessels (HBV), pseudopalisading cells around necrosis (PAN), and perinecrotic zone (PNZ) and was used to understand the histological importance of SPRY1 (Fig. 5D). Surprisingly, the expression of SPRY1 was significantly upregulated only in HBV and MVP. In contrast, SPRY1 expression was downregulated in LE, IT, CT, PNZ, and PAN tumor areas. The HBV and MVP regions showed the highest SPRY1 expression, which was correlated with angiogenesis and microvascular formation.

In conclusion, SPRY1 expression was associated with GSC self-renewal maintenance and GBM aggressiveness. Hence, we proposed that SPRY1 is a regulator of GBM stemness maintenance.

## 4 Discussions

In this study, we compared the mRNA expression of the *SPRY1* using the REMBRANDT dataset. Our findings showed that *SPRY1* expression is higher in human GBM than in normal brain tissues. Moreover, we showed that *SPRY1* is highly expressed in GSCs, while downregulated in its matched differentiated cells under serum-containing media. Interestingly, SPRY1 knockdown using siRNA in human GSCs decreases cell growth as well as a sphere-forming ability by repressing CD15 and CD133 expression, which have been used as markers for defining glioma stem cells.

We confirmed that high expression of SPRY1 is associated with patient poor survival, suggesting that SPRY1 may be a prognostic biomarker of GBM. To further evaluate the biological significance of SPRY1, we performed a KEGG pathway enrichment analysis. SPRY1 expression showed an association with genes that are related to focal adhesion, ECM-receptor interaction, and PI3K-Akt signaling pathways.

Sprouty 1 is encoded by the SPRY1 gene and has paradoxical roles in different tumors. It has a potential tumor-suppressive effect on prostate, ovarian, and lung cancers (Fritzsche et al., 2006, Masoumi-Moghaddam et al., 2014, Nemoto et al., 2011). However, increasing studies have suggested that it exerts an oncogenic effect in some cancers (Montico et al., 2020, He et al., 2016, Arceci, 2010, Terada et al., 2014). Qing et al. showed that SPRY1 has a selective function in a subset of EGFR expressed Triple-Negative Breast Cancer (TNBC) by promoting the aggressive phenotype *via* enhancing the EGF-mediated mesenchymal phenotype (He et al., 2016). SPRY1 was remarkably overexpressed in acute myeloid leukemia (AML) patients-derived cells and contributed to cell cycle progression and cell proliferation, suppressing cell apoptosis by activating the Hedgehog pathway (Lv et al., 2022). The Hedgehog pathway may modulate the number of cancer stem cells or the tumor microenvironment, such as leukemia and liver cancer (Pakula, 2019).

In primary GBM, EGFR signaling is a common genetic alteration and a major classical subtype signaling. ERFR overexpressing GBMs 50–60 % have EGFRvIII or ∆EGFR, which are common EGFR mutant forms. EGFR and EGFRvIII are involved in GBM invasion and angiogenesis, and EGFRvlll positive tumors have been correlated with low survival and poor prognosis [19]. Meanwhile, mouse SPRY1 can interact with E3 ubiquitin ligase c-Cbl (Wong et al., 2001). ﻿Usually, activated EGFR has phosphorylated tyrosines, that can be combined with c-Cbl. Bound activated EGFR undergoes degradation processes like ubiquitination, endocytosis, and subsequent degradation through the proteasomal/lysosomal pathways (Dikic and Giordano, 2003). SPRY1 phosphorylated on Tyr 55 can bind to c-Cbl, competing with activated EGFR for c-Cbl interaction. Consequently, the presence of SPRY1 suppressed EGFR ubiquitination, internalization, or degradation. As a result, EGFR relocated to the cell surface and maintained signaling activity (Christofori, 2003).

Our results showed that SPRY1 is correlated with GBM classical and mesenchymal subtypes and EGFR signaling. The results suggested that SPRY1 might interact with c-Cbl in GBM to promote GBM growth and aggressiveness. This study also showed that SPRY1 expression was closely associated with tumor angiogenesis. Based on these observations, we hypothesized that there is a region of neovascularization in the GBM microenvironment that expressed SPRY1 may contribute to regional regulation. With its important function in GBM progression, SPRY1 is a potential target for the development of new anti-angiogenic. SPRY1 has multiple functions depending on the interaction between molecules and tissues, and cell types.

Finally, our study showed that SPRY1 is highly expressed in GBM, and suppression of SPRY1 expression, decreased cell proliferation and tumorsphere formation. These findings suggest that SPRY1 plays an important role in cancer proliferation and stemness maintenance in GSCs.

## Financial disclosure

This research was supported by Basic Science Research Program through the 10.13039/501100003725National Research Foundation of Korea (NRF) funded by the Ministry of Education, Science andTechnology (no. NRF-2019R1I1A3A01059211).

## CRediT authorship contribution statement

Study Design: **Seo-Young Park, Hang Yeon Jeong, Sung-Hak Kim**, Data Collection: **Seo-Young Park, Hang Yeon Jeong**, Statistical Analysis: **Seo-Young Park**, **Hang Yeon Jeong**, Data Interpretation: **Seo-Young Park**, Manuscript Preparation: **Seo-Young Park, Don Carlo Batar**a, Literature Search: **Seo-Young Park**, Funds Collection: **Sung-Hak Kim**.

## Conflicts of Interest

The authors declare that they have no conflicts of interest.
